# Incidence of Secondary Hemophagocytic Lymphohistiocytosis in Critically-Ill COVID-19 Patients

**DOI:** 10.7759/cureus.16735

**Published:** 2021-07-29

**Authors:** Jamie Allen, Matthew M McCambridge, Hope Kincaid, Joshua A Kalter

**Affiliations:** 1 Department of Emergency and Hospital Medicine, Lehigh Valley Health Network Lehigh Valley Campus and University of Southern Florida Morsani College of Medicine, Lehigh Valley, USA; 2 Office of the Chief Medical Officer, Lehigh Valley Health Network Lehigh Valley Campus and University of Southern Florida Morsani College of Medicine, Lehigh Valley, USA; 3 Network Office of Research and Innovation, Lehigh Valley Health Network Lehigh Valley Campus and University of Southern Florida Morsani College of Medicine, Lehigh Valley, USA

**Keywords:** covid-19, sars-cov-2 (severe acute respiratory syndrome coronavirus -2), severe respiratory distress syndrome, adult respiratory distress syndrome, multi-viral pneumonia, retrospective studies, incidence and prognosis

## Abstract

Objective

Coronavirus disease 2019 (COVID-19) is associated with diffuse lung injury that can progress to acute respiratory distress syndrome, multisystem-organ failure, and death. The inflammatory storm seen in many COVID-19 patients closely resembles secondary hemophagocytic lymphohistiocytosis (sHLH) which has been described in other virus-associated severe sepsis. We sought to describe the incidence of sHLH in COVID-19 infected patients.

Design

In this retrospective study, we reviewed the records of critically ill COVID-19 positive patients to determine the incidence of sHLH. An H-score for sHLH diagnosis was determined for each study participant, with a score greater than 169 points needed for diagnosis.

Setting

A quaternary referral center in suburban Pennsylvania, USA.

Patients

All study participants had a positive COVID-19 test, and were deemed critically ill defined as receiving invasive mechanical ventilation and/or who expired.

Measurements and Main Results

Of the 246 records identified, 242 records met inclusion criteria and were reviewed. Eighty five patients were excluded from analysis due to missing H-score data parameters. Overall, 32 of 157 (20.38%, 95% CI:14.38-27.54%) patients met diagnostic criteria for sHLH. The average age was 69.42 years (standard deviation (SD) 14.81). Patients diagnosed with sHLH were more likely to be younger (61.09 years vs 69.38 years, *P* = 0.0036), male (71.88% vs 52.00%, *P* = 0.0433), and require mechanical ventilation (96.88% vs 72.80%, *P* = 0.0035).

Conclusions

Among critically ill COVID-19 positive patients, the incidence of sHLH is higher than previously reported in patients with non-COVID-19 related sepsis. Clinicians caring for COVID-19 patients should consider this secondary diagnosis and subsequent appropriate treatments, especially in those requiring mechanical ventilation.

## Introduction

Severe acute respiratory syndrome coronavirus 2 (SARS-CoV-2), the cause of coronavirus disease 2019 (COVID-19), is a novel beta coronavirus that emerged from a zoonotic source in Wuhan, China in 2019 [1]. COVID-19 can present in a wide spectrum of clinical symptoms; the majority of COVID-19 cases are asymptomatic or present with a mild viral prodrome of fever, cough, dyspnea, and myalgias [2]. Some patients progress to viral pneumonia requiring hospitalization. A relatively small percentage of patients progress to acute respiratory distress syndrome, multisystem-organ failure, and death [3,4].

In many of very severe cases, evidence of cytokine storm has been demonstrated through increased acute-phase reactants and inflammatory modulators. Significant elevation of ferritin, Interleukin (IL) 6, C-reactive protein, IL-1, Granulocyte-macrophage colony stimulating factor (GMCSF), and tumor necrosis factor-alpha (TNF-α) can be seen [3]. Immunosuppressive therapy with glucocorticoids has been shown to provide benefit in severely ill patients with COVID-19 disease [4]. Mehta et al. proposed that the clinical profile of COVID-19 patients closely resembled that of sHLH, with hyperferritinemia, hypertriglyceridemia, cytopenias, fever, organomegaly, transaminitis, hypofibrinogenemia, and high IL-6 [5].

Secondary hemophagocytic lymphohistiocytosis is a syndrome that results from defective hyperactivation of natural killer and cytotoxic T-cells that can lead to multi-organ failure similar to what some patients have been experiencing with COVID-19 infection [6,7]. Secondary hemophagocytic lymphohistiocytosis has many known triggers including neoplasm, autoimmune, and infection, with the most common infectious cause being viral [7]. In septic patients, the incidence of sHLH has been estimated between 3.7 and 4.3% [7]. Prompt treatment of sHLH with broad immunosuppressive or immunomodulatory therapies such as glucocorticoids, cyclosporine, intravenous gamma globulin, anti-IL-6 and anti-IL-1 agents, or chemotherapeutics is important for favorable outcomes. However, use of immunosuppressant therapy in COVID-19 patients must be done cautiously, as co-infections can occur with COVID-19, and such infections have been associated with severity of SARS-CoV-2 as well as poor outcomes [8]. 

SHLH can be diagnosed by calculating an “H-score” consisting of multiple data points. The H-score generates a probability for the presence of sHLH, and according to Fardet et al., a score greater than 169 points is 93% sensitive and 86% specific for sHLH [9]. While the presence of bone marrow hemophagocytosis on aspirate is also a criterion in the H-score, it is not mandatory for a diagnosis and is often not practical to obtain in critically ill patients [6].

While the role of cytokine storm in the pathogenesis of some severe COVID-19 infections has been discussed within the literature, to our knowledge, the incidence of sHLH in these patients has not been determined. Here we present a retrospective review to determine the incidence of sHLH in critically ill, COVID-19 positive patients at a single institution in northeastern Pennsylvania, USA.

## Materials and methods

We retrospectively reviewed the records of 246 critically ill, COVID-19 positive patients to determine the incidence of sHLH. We defined critically ill as any patient requiring invasive mechanical ventilation and/or who expired. Patients were identified using an internal COVID-19 registry automatically populated by a positive test. Laboratory work, medical history, vital signs, and imaging (computed tomography (CT) scan and/or ultrasound) were reviewed to calculate an H-score for as many study participants as possible. 

Patients had to meet the following inclusion criteria: admission to our hospital network between March 15th and June 15th, 2020, positive COVID-19 test upon or during admission, age ≥ 18 years, having received invasive mechanical ventilatory support related to COVID-19 infection and/or death attributed to COVID-19 infection. We further excluded any COVID-19 positive patient with baseline cytopenia (defined as platelets ≤ 110,000 per mm^3^, white blood cell ≤5.0 g/dL, and/or hemoglobin ≤ 9.2 g/dL).

We identified 246 patients, and 242 met all entry criteria. Three of the four excluded had one or more baseline cytopenia and the remaining patient underwent invasive mechanical ventilation for a surgical procedure rather than for respiratory support related to COVID-19 infection. 

All data were obtained by a single reviewer from patients’ electronic medical records. We collected data on temperature, presence or absence of hepatomegaly and splenomegaly, triglycerides, fibrinogen, ferritin, serum aspartate aminotransferase, presence/absence of immune suppression (defined as known Human Immunodeficiency Virus (HIV) infection or those receiving long-term immunosuppression therapy), date of admission and discharge, age, ethnicity, sex, blood type, hospital, and Intensive Care Unit (ICU) length of stay (LOS), ventilator days, and discharge disposition. No study participant underwent bone marrow aspirate testing. Study data were collected and managed using REDCap electronic data capture tools hosted at Lehigh Valley Health Network. REDCap (Research Electronic Data Capture) is a secure, web-based software platform designed to support data capture for research studies, providing 1) an intuitive interface for validated data capture; 2) audit trails for tracking data manipulation and export procedures; 3) automated export procedures for seamless data downloads to common statistical packages; and 4) procedures for data integration and interoperability with external sources [10,11]. Lehigh Valley Health Network's Institutional Review Board approved Study 00000670 on 7/23/2020. 

Descriptive statistics were generated for all variables collected. The mean was presented with the standard deviation for normally distributed continuous variables. Categorical variables were presented as frequencies and percentages. Variables were then compared between groups. The chi-square test of independence was used for categorical variables unless > 20% of the expected cell counts were < 5 in which case the Fisher’s exact test was used instead. The independent samples t-test was used for continuous variables; however, if the data in either group was not normally distributed the Mann-Whitney U test was used instead. The incidence of sHLH was calculated excluding those who did not have sufficient data to accurately determine H-score. The confidence limits around the incidence were calculated using the exact binomial method. All statistical tests were two-tailed and a P-value <0.05 was considered statistically significant. The data analysis for this paper was generated using SAS software, Version 9.4 (SAS Institute Inc., Cary, NC, USA).

## Results

Of the 246 records identified, 242 were reviewed (Table 1). Eighty-five patients were excluded from the sHLH incidence calculation due to a lack of data to determine the presence or absence of sHLH. Specifically, those for whom the sum of the maximum value of the missing H-score parameters could potentially raise the score above the 169-point cutoff. The average age was 69.42 years (standard deviation (SD) 14.80). One hundred forty-two patients were male (58.68%) and 100 were female (41.32%). Most patients were white (62.40%) followed by other (31.40%), black (3.31%), and Asian/Pacific Islander (2.89%). Patients diagnosed with sHLH were more likely to be younger (61.09 years vs 69.48 years, P = 0.0036) and male (71.88% vs 52.00%, P = 0.0433). We found no statistically significant association between sHLH and race (P = 0.8860). 

**Table 1:** Demographics Abbreviations: sHLH = secondary Hemophagocytic Lymphohistiocytosis, SD = Standard Deviation. Notes: Numbers presented in the table were calculated for column totals. The cut-point for sHLH was an H-score of >169. Some patients were missing parameters used to calculate H-Score. If the sum of the maximum value of the missing parameters could have potentially pushed the patient over the cut-point for sHLH their final H-score was marked invalid and the presence of sHLH could not be determined (these patients are represented in the second to last column in the table). a. P-values were computed for the comparison between those with and without sHLH, those shown in bold are statistically significant. b. Calculated using the independent samples t-test. c. Calculated using the chi-square test. d. Calculated using Fisher’s exact test. e. 110 records were missing data for blood type so n=132.

	Entire Sample (n=242)	sHLH (n=32)	No sHLH (n=125)	Missing Parameters (n=85)	P-value^a^
Age mean±SD	69.42±14.81	61.09±14.65	69.38±14	72.61±14.96	0.0036^b^
Gender n(%)					0.0433^c^
Male	142 (58.68)	23 (71.88)	65 (52)	54 (63.53)	
Female	100 (41.32)	9 (28.13)	60 (48)	31 (36.47)	
Race n(%)					0.8860^d^
White	151 (62.40)	20 (62.50)	75 (60)	56 (65.88)	
Black	8 (3.31)	1 (3.13)	4 (3.20)	3 (3.53)	
Asian/Pacific Islander	7 (2.89)	2 (6.25)	5 (4)	0	
Other	76 (31.40)	9 (28.13)	41 (32.80)	26 (30.59)	
Blood Type n(%)^e^					0.6207^d^
A-	5 (3.79)	0	3 (4.11)	2 (5.88)	
B-	4 (3.03)	1 (4)	2 (2.74)	1 (2.94)	
A+	43 (32.58)	6 (24)	24 (32.88)	13 (38.24)	
B+	10 (7.58)	2 (8)	7 (9.59)	1 (2.94)	
AB-	-	-	-	-	
AB+	3 (2.27)	2 (8)	1 (1.37)	0	
O+	60 (45.45)	13 (52)	32 (43.84)	15 (44.12)	
O-	7 (5.30)	1 (4)	4 (5.48)	2 (5.88)	

A total of 157 patients had sufficient data to calculate an H-score, and among them, the incidence of sHLH was 20.38% (95% CI: 14.38 - 27.54%). Eight other patients had an H-score between 160-169 points (Table 2).

**Table 2:** H-score Parameters Abbreviations: sHLH = secondary Hemophagocytic Lymphohistiocytosis, SD = Standard Deviation, Temp = temperature, Hepat = Hepatomegaly, Splen = Splenomegaly, and AST = Serum Glutamic Oxaloacetic Transaminase. Notes: Numbers presented in the tables were calculated for column totals. The cut-point for sHLH was an H-score of >169. Median and Interquartile range (IQR) are presented for temperature, hemoglobin, platelets, leukopenia, and H-score because the data was significantly skewed for at least one of the groups. The cut-point for anemia was hemoglobin of ≤ 9.2 (g/dL). Cut-point for thrombocytopenia was a platelet count of ≤ 110 (#/L). The cut-point for leukopenia was a white blood cell count ≤ 5.0 (g/dL). Some patients were missing parameters used to calculate H-score. If the sum of the maximum value of the missing parameters could have potentially pushed the patient over the cut-point for sHLH their final H-score was marked invalid and the presence of sHLH could not be determined (these patients are represented in the second to last column in the table). a. P-values were computed for the comparison between those with and without sHLH, those shown in bold are statistically significant. b. Three records were missing for immunosuppression so n=239. c. Calculated using Fisher’s exact test. d. Calculated using the Mann-Whitney U test. e. Calculated using the chi-square test. f. 126 records were missing for organomegaly so n=116. g. One record was missing for hemoglobin/anemia, platelets/thrombocytopenia, leukopenia, and AST so n=241 for those variables. h. 77 records were missing for cytopenia so n=165. i. 29 records were missing for ferritin so n=213. j. 70 records were missing for triglyceride so n=172. k. 91 records were missing for fibrinogen so n =151. l. Due to missing parameters H-score was unable to be calculated for 85 records so n for H-score is 157.

	Entire Sample (n=242)	sHLH (n=32)	No sHLH (n=125)	Missing Parameters (n=85)	P-value^a^
Immunosuppression n(%)^b^					0.6967^c^
Yes	14 (5.86)	3 (9.38)	8 (6.40)	3 (3.66)	
No	225 (94.14)	29 (90.63)	117 (93.60)	79 (96.34)	
Temp (°F) median (IQR)	101.65 (100.20-102.80)	103.15 (102.50-103.55)	101.20 (100.20-102.30)	101.40 (99.10-102.70)	<0.0001^d^
Temp (°F) n(%)					<0.0001^e^
< 101.1	95 (39.26)	1 (3.13)	60 (48)	34 (40)	
101.1-102.9	93 (38.43)	11 (34.38)	50 (40)	32 (37.65)	
>102.9	54 (22.31)	20 (62.50)	15 (12)	19 (22.35)	
Organomegaly n(%)^f^					<0.0001^c^
No	80 (68.97)	6 (25)	51 (75)	23 (95.83)	
Hepat OR Splen	31 (26.72)	15 (62.50)	16 (23.53)	0	
Hepat AND Splen	5 (4.31)	3 (12.50)	1 (1.47)	1 (4.17)	
Hemoglobin median (IQR)^g^	9 (7.80-12.10)	7.55 (7-8.30)	8.80 (7.70-11.70)	11.20 (8.80-12.85)	0.0003^d^
Anemia n(%)^g^					0.0137^e^
Yes	127 (52.70)	26 (81.25)	72 (57.60)	29 (34.52)	
No	114 (47.30)	6 (18.75)	53 (42.40)	55 (65.48)	
Platelets (#/L) median (IQR)^g^	167 (102-253)	123 (85-170)	173 (102-262)	195.50 (117-273.50)	0.0072^d^
Thrombocytopenia n(%)^g^					0.1390^e^
Yes	64 (26.56)	13 (40.63)	34 (27.20)	17 (20.24)	
No	177 (73.44)	19 (59.38)	91 (72.80)	67 (79.76)	
Leukopenia (g/dL) median (IQR)^g^	6.70 (4.30-12)	4.75 (3.85-6.70)	6.70 (4.30-10.40)	8.50 (5.15-13.60)	0.0351^d^
Leukopenia n(%)^g^					0.0012^e^
Yes	75 (31.12)	19 (59.38)	36 (28.80)	20 (23.81)	
No	166 (68.88)	13 (40.63)	89 (71.20)	64 (76.19)	
Cytopenia n(%)^h^					0.0043^e^
1 lineage	82 (49.70)	8 (26.67)	58 (61.05)	16 (40)	
2 lineages	66 (40)	16 (53.33)	28 (29.47)	22 (55)	
3 lineages	17 (10.30)	6 (20)	9 (9.47)	2 (5)	
Ferritin (ng/mL) n(%)^i^					<0.0001^e^
< 2,000	138 (64.79)	2 (6.25)	102 (83.61)	34 (57.63)	
2,000-6,000	53 (24.88)	18 (56.25)	17 (13.93)	18 (30.51)	
> 6,000	22 (10.33)	12 (37.50)	3 (2.46)	7 (11.86)	
Triglyceride (mg/dL) n(%)^j^					<0.0001^e^
< 150	42 (24.42)	0	39 (35.78)	3 (9.68)	
150-400	88 (51.16)	13 (40.63)	60 (55.05)	15 (48.39)	
> 400	42 (24.42)	19 (59.38)	10 (9.17)	13 (41.94)	
Fibrinogen (mg/dL) n(%)^k^					0.3605^c^
≤ 250	9 (5.96)	3 (11.11)	5 (4.81)	1 (5)	
> 250	142 (94.04)	24 (88.89)	99 (95.19)	19 (95)	
AST (U/L) n(%)^g^					0.5826^c^
< 30	9 (3.73)	0	4 (3.20)	5 (5.95)	
≥ 30	232 (96.27)	32 (100)	121 (96.80)	79 (94.05)	
Final H-Score median (IQR)^l^	107 (52-160)	190 (183.50-195)	87 (43-119)	-	<0.0001^d^

We found no significant difference between sHLH and non-sHLH patients in relation to hospital LOS (18 vs 15 days, P = 0.0704), ICU LOS (16 vs 14 days, P = 0.2053), ventilator days (14 vs 13 days, P = 0.2761), or discharge disposition (P = 0.5159) (Table 3). Patients with sHLH were significantly more likely to require mechanical ventilation (96.88% vs 72.80%, P = 0.0035).

**Table 3:** Hospitalization Information Abbreviations: sHLH = secondary Hemophagocytic Lymphohistiocytosis, LOS = Length of Stay, IQR = Interquartile Range, ICU = Intensive Care Unit, Vent = ventilator, DC = discharge, Dispo = disposition, Rehab/SNF = Rehabilitation/Skilled Nursing Facility. Notes: Numbers presented in the table were calculated for column totals. Cut-point for sHLH was a H-score of >169. Median and IQR are presented for LOS, ICU LOS, and Vent Days because the data was significantly skewed to the right. Some patients were missing parameters used to calculate H-Score. If the sum of the maximum value of the missing parameters could have potentially pushed the patient over the cut-point for sHLH their final H-Score was marked invalid and presence of sHLH could not be determined (these patients are represented in the second to last column in the table). a. P-values were computed for the comparison between those with and without sHLH, those shown in bold are statistically significant. b. Calculated using the Mann-Whitney U test. c. N for ICU LOS is 198 because other 44 patients were not admitted to ICU during hospitalization. d. Calculated using the chi-square test. e. N for vent days is 167 because the other 75 patients were not on a vent during hospitalization f. Calculated using the Fisher’s exact test.

	Entire Sample (n=242)	sHLH (n=32)	No sHLH (n=125)	Missing Parameters (n=85)	P-value^a^
LOS median(IQR)	12 (5-23)	18 (8.50-35.50)	15 (6-24)	6 (2-15)	0.0704^b^
ICU LOS median (IQR)^c^	12 (6-20)	16 (8.50-29.50)	14 (8-20)	8 (5-16)	0.2053^b^
Vent n(%)					0.0035^d^
Yes	167 (69.01)	31 (96.88)	91 (72.80)	45 (52.94)	
No	75 (30.99)	1 (3.13)	34 (27.20)	40 (47.06)	
Vent Days median(IQR)^e^	12 (6-20)	14 (8-30)	13 (6-21)	8 (5-15)	0.2761^b^
DC Dispo n(%)					0.5159^f^
Deceased	167 (69.01)	19 (59.38)	83 (66.40)	65 (76.47)	
Rehab/SNF	70 (28.93)	13 (40.63)	39 (31.20)	18 (21.18)	
Home	5 (2.07)	0	3 (2.40)	2 (2.35)	

## Discussion

Our study suggests the incidence of sHLH in critically ill, COVID-19 patients is higher than previously cited in the literature for non-COVID-19 related sepsis [7]. Our sHLH patients were significantly younger suggesting profound hyperinflammation requires a functional immune system. Inflammation is an essential part of the immune response, but in patients with COVID-19, there is an excessive and prolonged cytokine storm, leading to acute respiratory distress syndrome, multiple-organ failure, and death [12]. Thirty-one of the thirty-two patients diagnosed with sHLH underwent mechanical ventilation during admission. The remaining patient was not mechanically ventilated in keeping with their pre-existing advanced directive.

To more accurately reflect true incidence, we included patients with missing data in our analysis if the sum of the maximum value of the missing H-score parameters were unable to eclipse the 169-point threshold. 

The mainstay of sHLH treatment is immunosuppression, and the recently published RECOVERY trial demonstrated improved outcomes for COVID-19 patients receiving dexamethasone with the greatest benefit in mechanically ventilated patients [9]. Eight patients with H-scores between 160-169 points all underwent mechanical ventilation. We believe these patients would have benefitted from immunosuppression despite not meeting the sHLH diagnostic threshold. Additionally, of the 85 patients with missing data excluded from incidence analysis, five would have surpassed the 169-point threshold with either the presence of hepatomegaly or splenomegaly. Five other patients would have met diagnostic criteria if they had both organomegalies. We believe this information demonstrates that the incidence in our study is likely underestimated and underscores the importance of considering immunosuppressive treatment in patients with a reactive hemophagocytic-like syndrome despite not meeting a true sHLH diagnosis. 

Our study had several limitations. A single researcher reviewed data retrospectively from one institution. Most of our study participants were white, raising concerns with external validity. Many patients had missing data, limiting our ability to calculate accurate H-scores for all participants. 

We attributed absent data to several factors. Some blood testing (namely triglyceride, fibrinogen, and ferritin levels) were not routinely obtained at our institution early in the pandemic. Our health network limited imaging for infection control purposes, and we could not comment on the presence/absence of organomegaly in many patients. Finally, no patient underwent bone marrow biopsy due to feasibility in critical illness. Other researchers have questioned the use of the H-score for this reason; the condition of patients with COVID-19 may not permit bone marrow examination, which can make it difficult to demonstrate hemophagocytosis [13]. Furthermore, some researchers recommend against using the H-score due to a possible lack of sensitivity; while hyperferritinemia is associated with sHLH, in severe COVID-19 patients, the ferritin concentrations often do not reach the required threshold of 2000·0 ng/mL until much later in the disease, thus limiting early intervention [14]. Expeditious management of the cytokine storm in the early stage is critical for improving treatment and reducing the mortality rate of COVID-19 patients [12].

In summary, among critically ill, COVID-19 positive patients, the incidence of sHLH is higher than previously reported in sepsis literature. Our COVID-19 positive critically ill sHLH patients were significantly younger suggesting profound hyperinflammation requires a functional immune system. Clinicians caring for COVID-19 patients should consider this secondary diagnosis and appropriate treatments. They should especially review this for COVID-19 positive patients requiring mechanical ventilation.

## Conclusions

In summary, among critically ill COVID-19 positive patients, the incidence of sHLH is higher than previously reported in sepsis literature. Our COVID-19 positive critically ill sHLH patients were significantly younger suggesting profound hyperinflammation requires a functional immune system. Clinicians caring for COVID-19 patients should consider this secondary diagnosis and appropriate treatments. They should especially review this for COVID-19 positive patients requiring mechanical ventilation.
